# Supplementary material for: Vertical two-dimensional layered fused aromatic ladder structure
Source: Nat Commun. 2020 Apr 24;11:2021. doi: 10.1038/s41467-020-16006-0 (PMC7181601; doi:10.1038/s41467-020-16006-0)
Supplement: Supplementary file 1 — Supplementary Information [file 41467_2020_16006_MOESM1_ESM.pdf]

Supplementary Information

Vertical two-dimensional layered fused aromatic ladder structure

Noh *et al.*

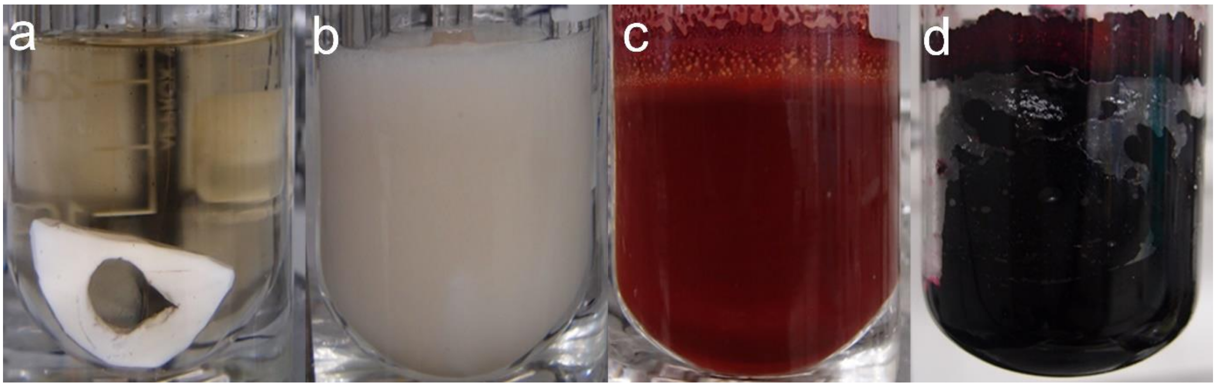

Supplementary Figure 1 | Photographs taken during reaction of the V2D-BBL structure. **a**, After the dehydrochlorination of triptycene hexamine (THA) hexahydrochloride in PPA at room temperature, a transparent solution was observed, suggesting complete dehydrochlorination. **b**, After a second monomer (NDA) was added at room temperature, **c**, polymerized at 150 °C before cyclization, **d**, polymerized at 175 °C, a gel-type viscous dark solution was formed, indicating cyclization had occurred.

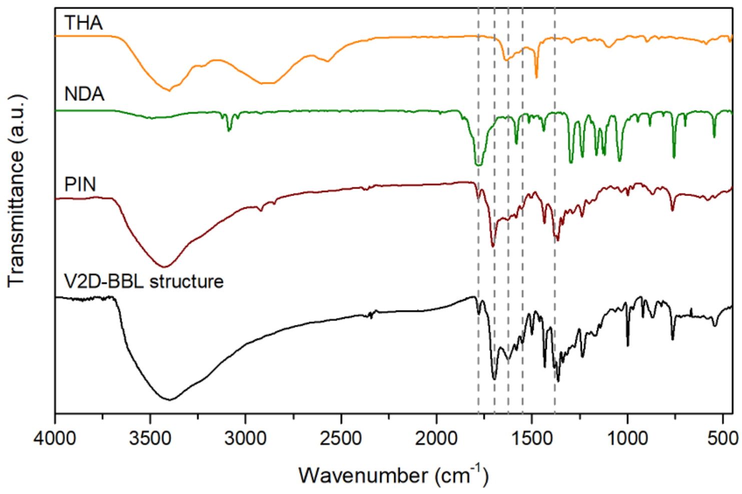

Wavenumber (cm^{-1})	Functional group
1628	C=N stretch
1387	C-N-C stretch
1698 (imide), 1706 (BBL)	Carbonyl C=O stretch
1552	Aromatic C=C stretch
1780	Unreacted C=O stretch

Supplementary Figure 2│FT-IR spectra (KBr pellets) of triptycene hexamine (THA) hexahydrochloride (orange line), NDA (green line), the PIN (wine line) and V2D-BBL structure (black line), confirming the benzimidazole ring.

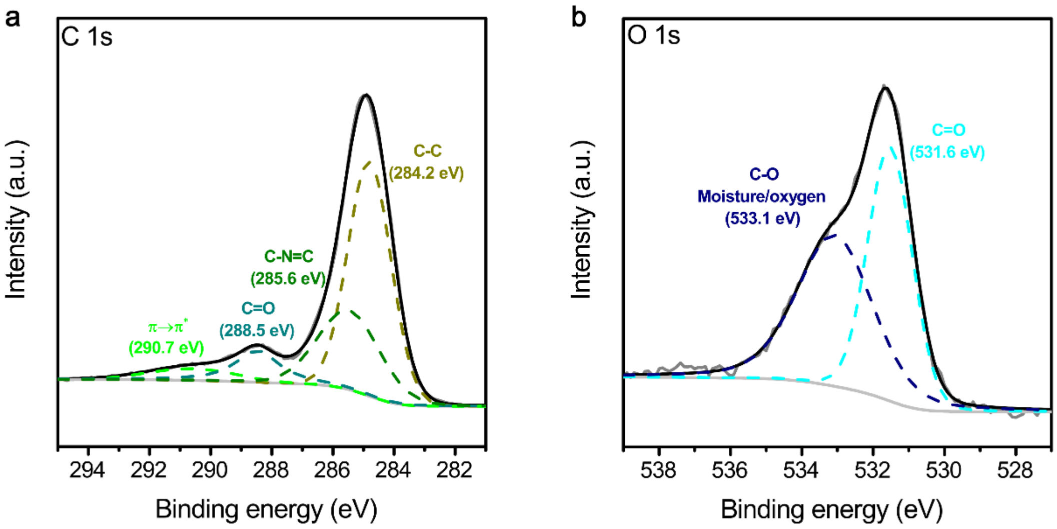

Supplementary Figure 3 | High-resolution XPS spectra of the V2D-BBL structure of **a**, C 1s and **b**, O 1s.

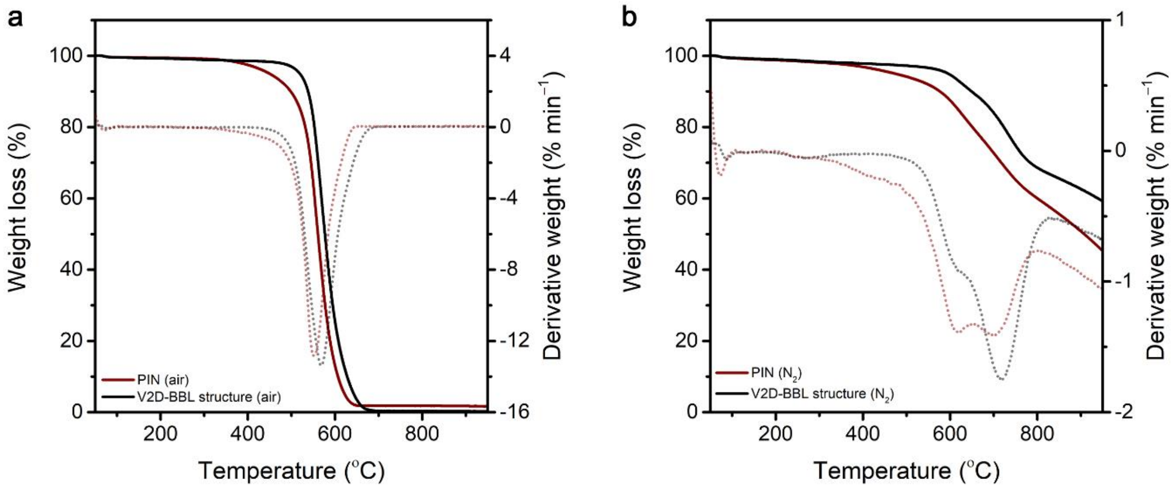

Supplementary Figure 4 | TGA curves of the PIN (wine line) and the V2D-BBL structure (black line) under **a**, air and **b**, nitrogen atmospheres.

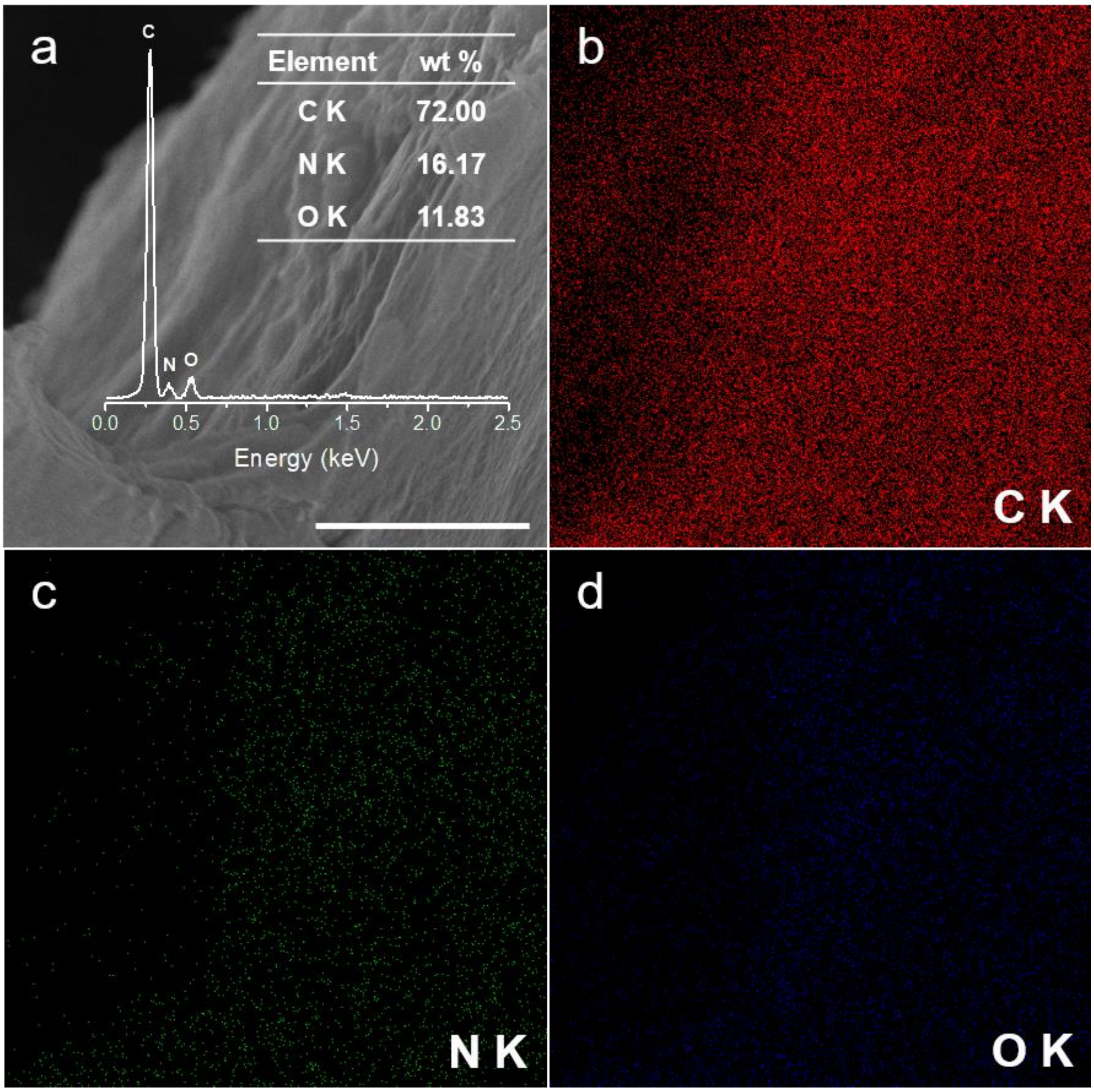

Supplementary Figure 5 | a, SEM image with EDS spectrum and elemental compositions of V2D-BBL structure. Scale bar: 2 μm. Corresponding elemental mappings: **b**, carbon, **c**, nitrogen and **d**, oxygen.

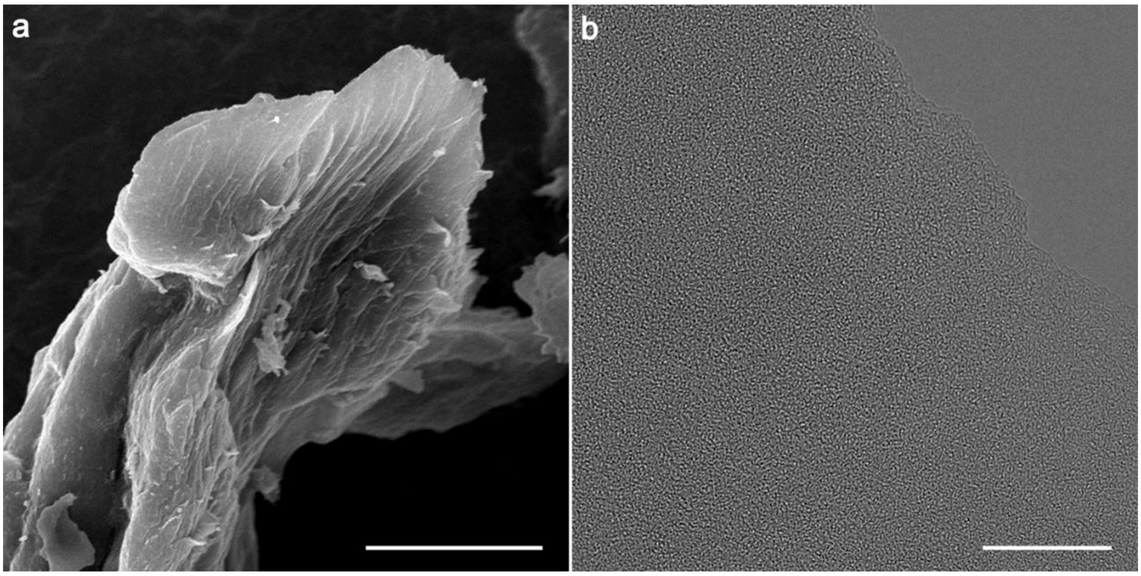

Supplementary Figure 6 | a, SEM image and **b**, HR-TEM image of the V2D-BBL structure. Scale bars: **a**, 5 μm; **b**, 20 nm.

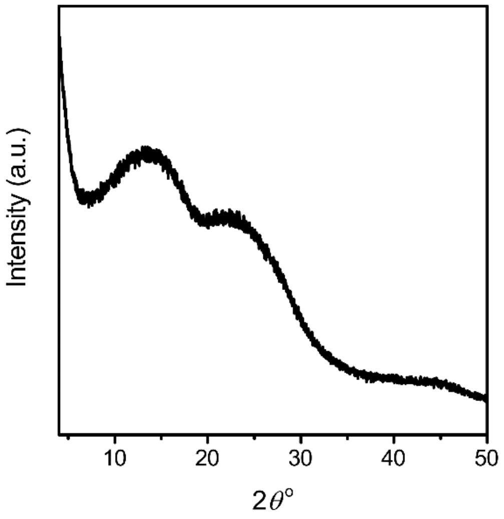

**Supplementary Figure 7 | **PXRD pattern of the V2D-BBL structure.

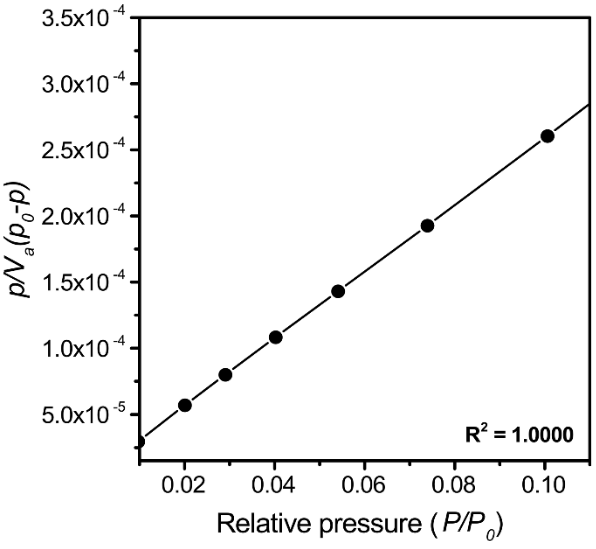

**Supplementary Figure 8 | **BET linear plot of the V2D-BBL structure obtained from N_2 adsorption and desorption isotherm at 77 K.

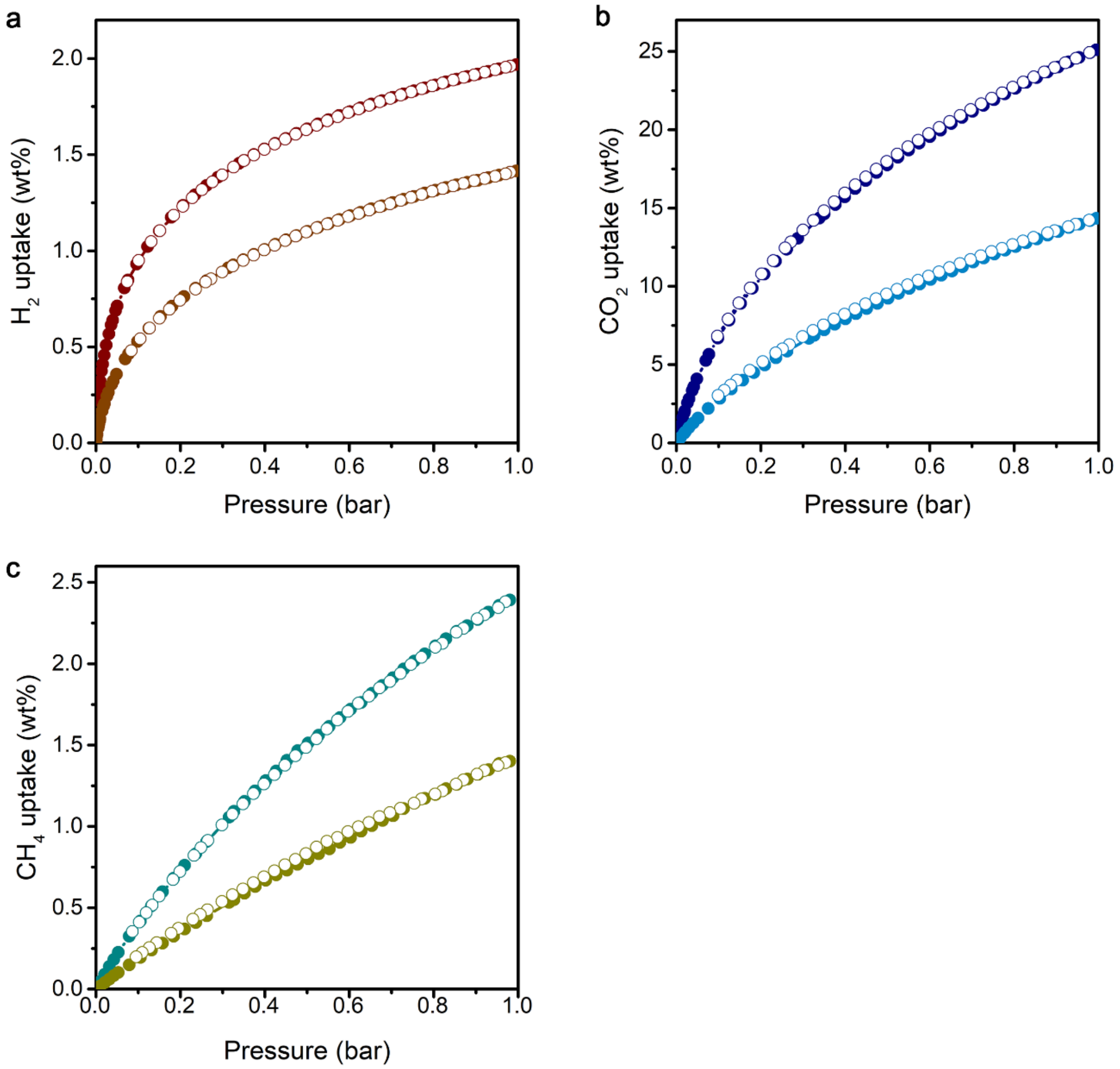

Supplementary Figure 9 | Low-pressure gas uptake isotherms. a, H_2 adsorption-desorption isotherms of the V2D-BBL structure measured at 77 K (wine) and 87 K (brown). **b**, CO_2 uptake isotherms measured at 273 K (navy) and 298 K (sky blue). **c**, CH_4 uptake isotherms measured at 273 K (dark cyan) and 298 K (dark yellow) in weight percent scale.

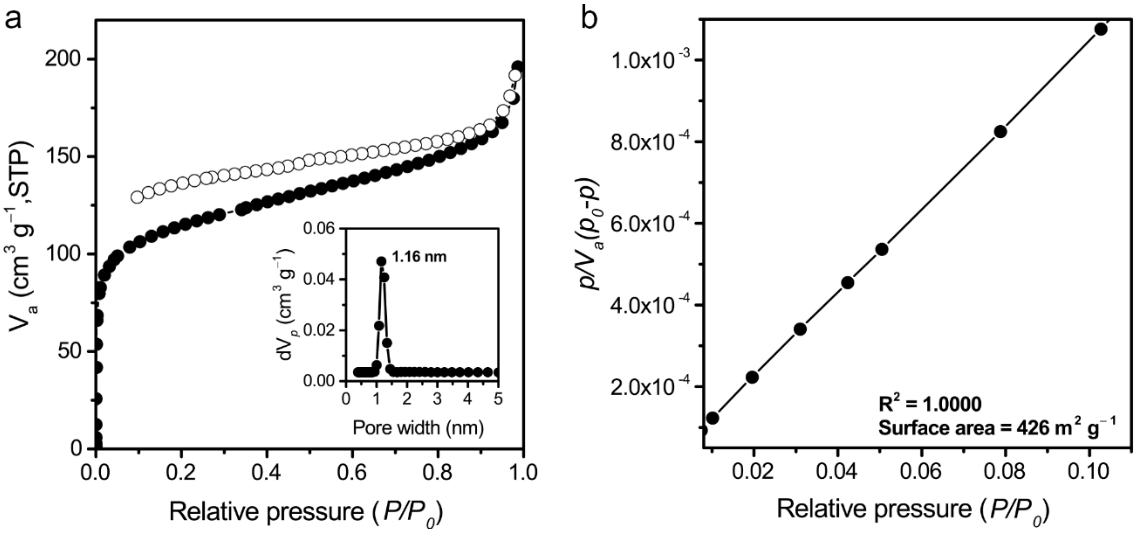

Supplementary Figure 10 | a, N_2 adsorption and desorption isotherm of the PIN sample measured at 77 K. Inset: corresponding NLDFT pore size distribution. **b**, BET linear plot of the PIN obtained from N_2 isotherm at 77 K.

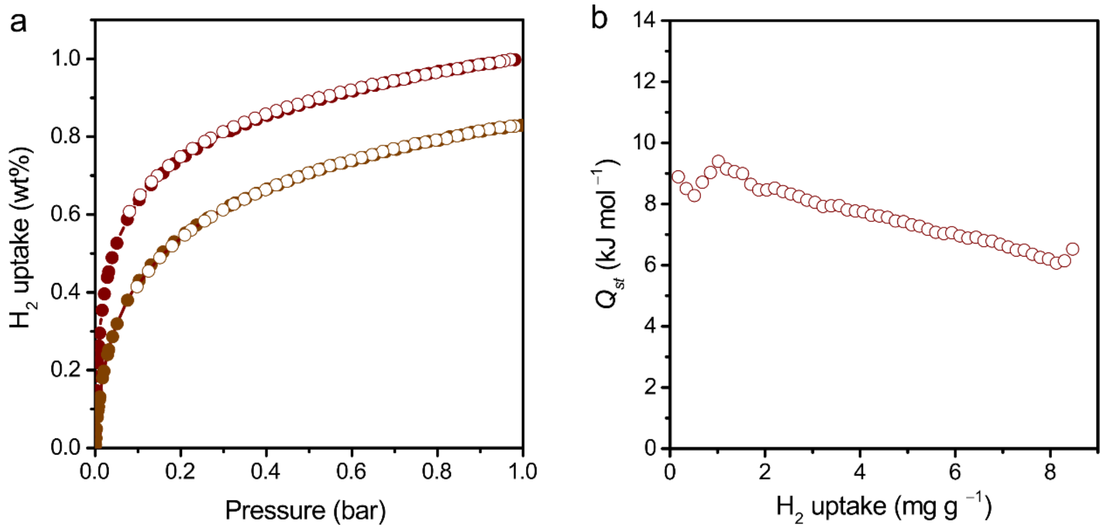

Supplementary Figure 11 | a, H_2 adsorption and desorption isotherms of the PIN measured at 77 K (wine line) and 87 K (brown) of the PIN. **b**, Q_{st} graph as a function of gas loading calculated from low-pressure isotherms at 77 and 87 K.

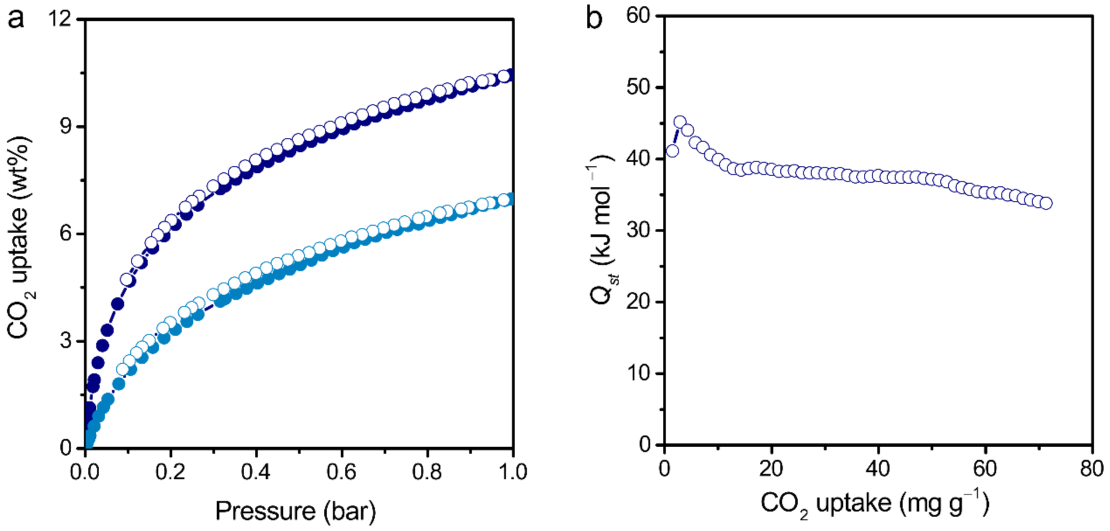

Supplementary Figure 12 | a, CO_2 adsorption and desorption isotherms of the PIN measured at 273 K (navy) and 298 K (sky blue). **b**, Q_{st} graph as a function of gas loading calculated from low-pressure isotherms at 273 and 298 K.

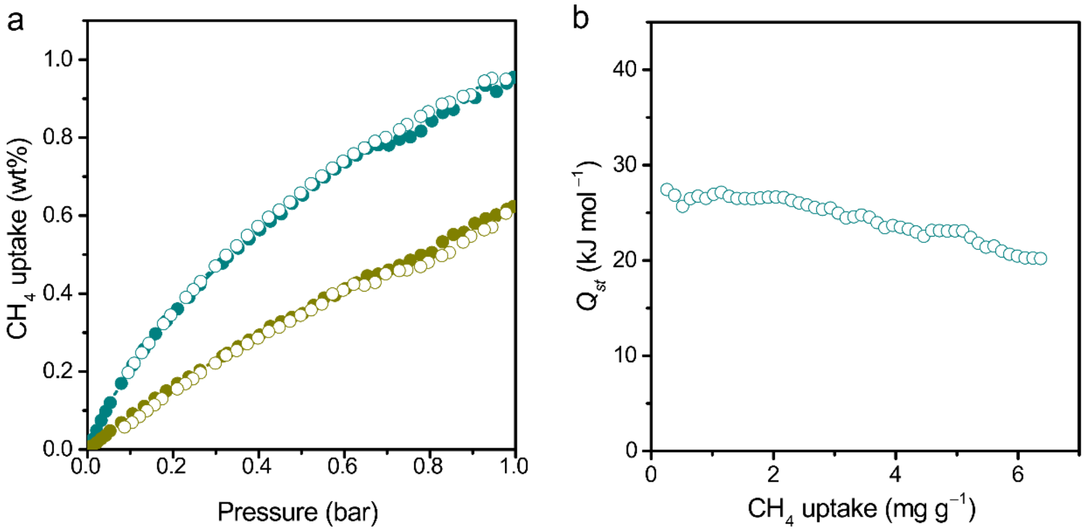

Supplementary Figure 13 | a, CH_4 adsorption and desorption isotherms of the PIN measured at 273 K (dark cyan) and 298 K (dark yellow) of the PIN. **b**, Q_{st} graph as a function of gas loading calculated from low-pressure isotherms at 273 and 298 K.

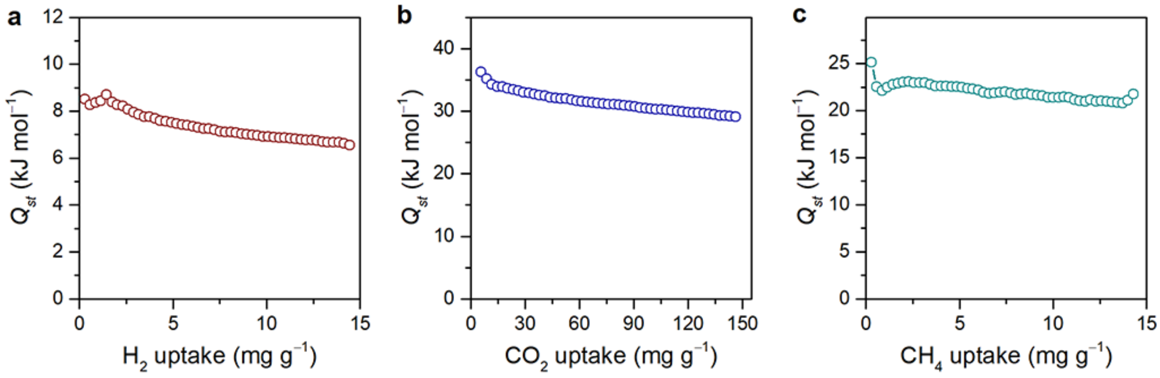

Supplementary Figure 14 | The isosteric heat of adsorption (Q_{st}) values of the V2D-BBL structure. a, Q_{st} for H_2 calculated from H_2 isotherms at 77 and 87 K. **b**, Q_{st} for CO_2 calculated from CO_2 isotherms at 273 and 298 K. **c**, Q_{st} for CH_4 calculated from CH_4 isotherms at 273 and 298 K.

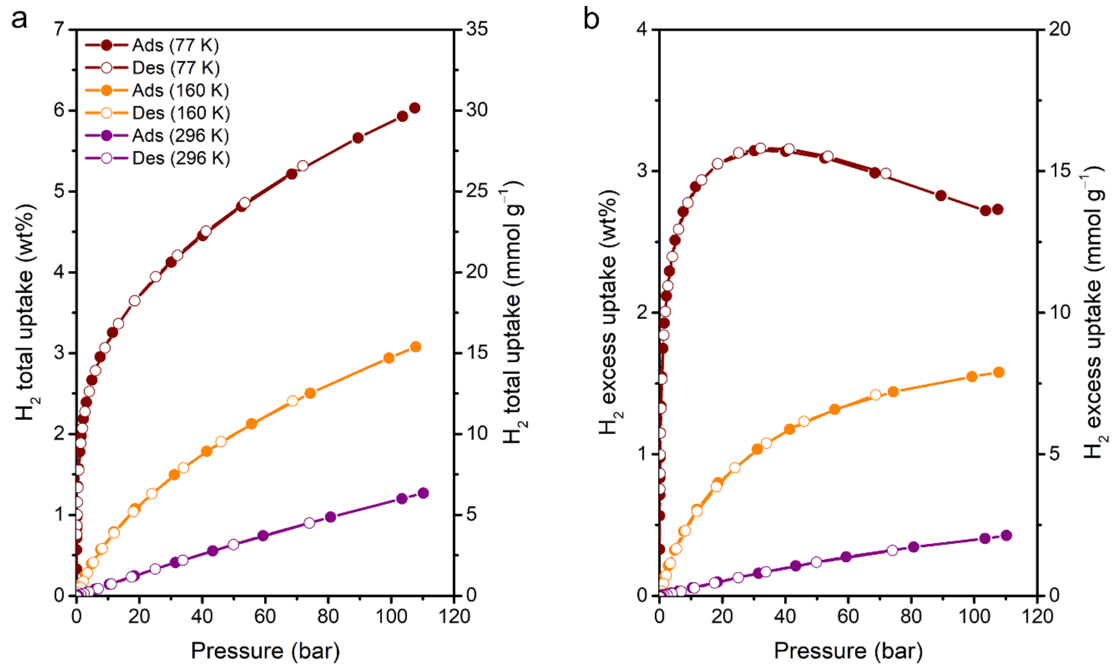

Supplementary Figure 15 | High-pressure H_2 adsorption and desorption isotherms of the V2D-BBL structure measured at 77 K (wine red), 160 K (orange) and 296 K (purple) at 110 bar for **a**, total uptake and **b**, excess uptake.

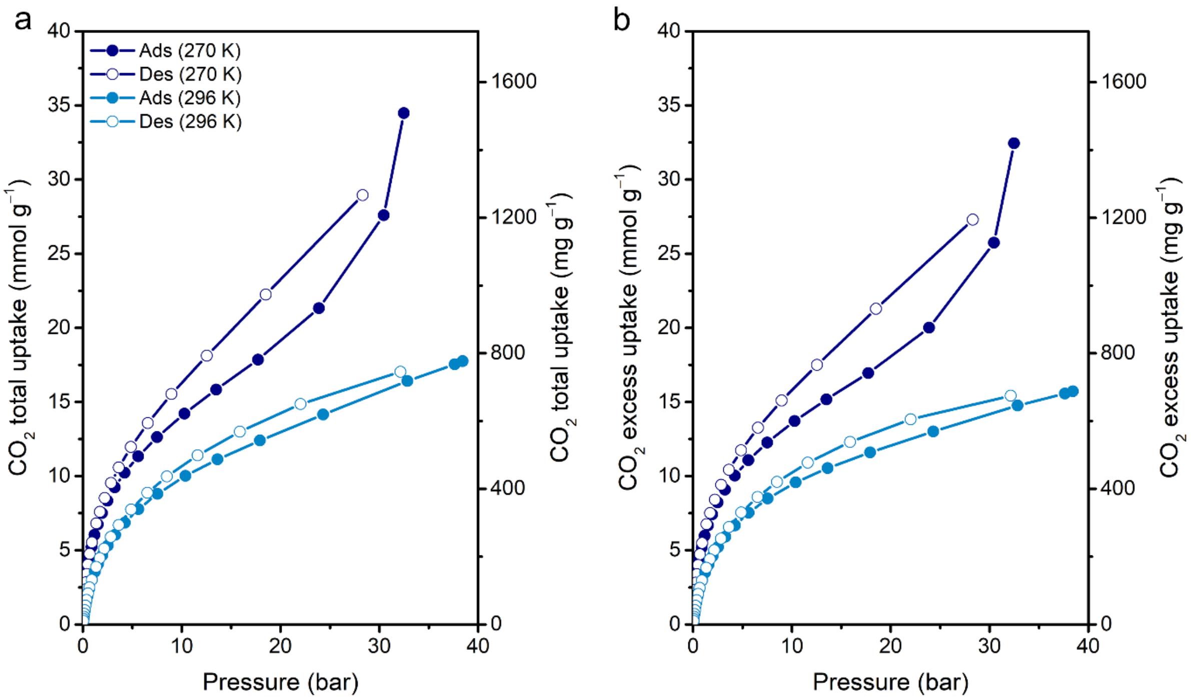

Supplementary Figure 16 | ** High-pressure CO_2 adsorption and desorption isotherms of the V2D-BBL structure measured at 270 K (navy) and 296 K (sky blue) up to 40 bar for **a, total uptake and **b**, excess uptake.

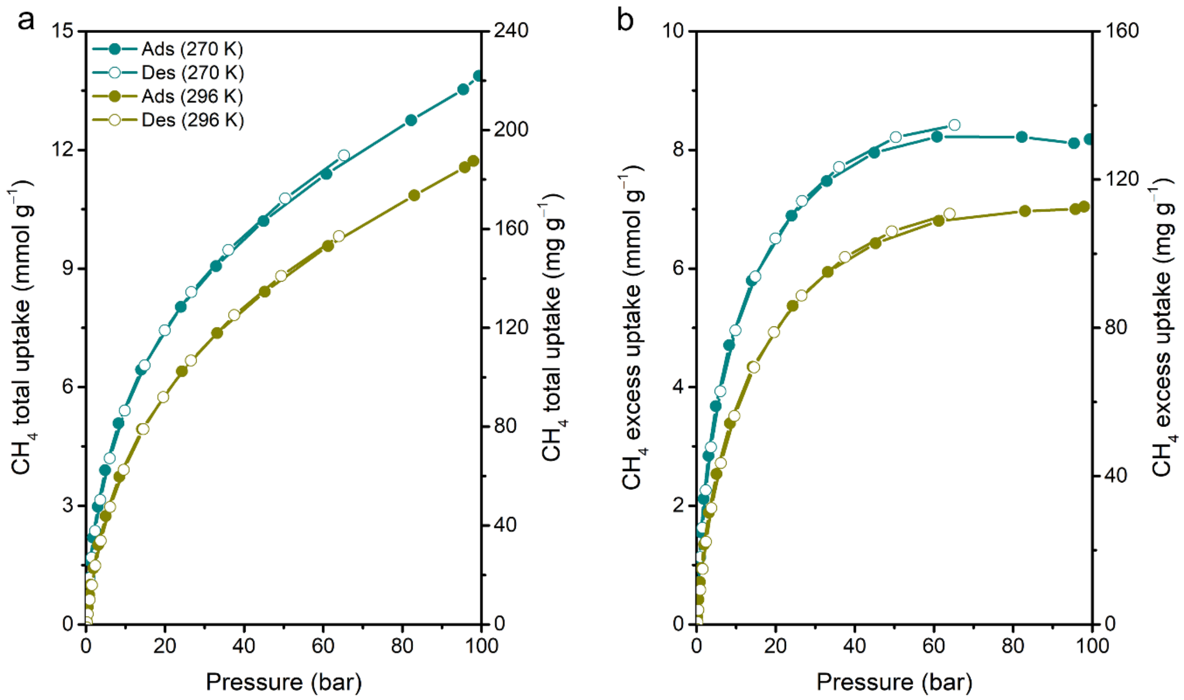

Supplementary Figure 17 | High-pressure CH_4 adsorption and desorption isotherms of the V2D-BBL structure measured at 270 K (dark cyan) and 296 K (dark yellow) at 100 bar for **a**, total uptake and **b**, excess uptake.

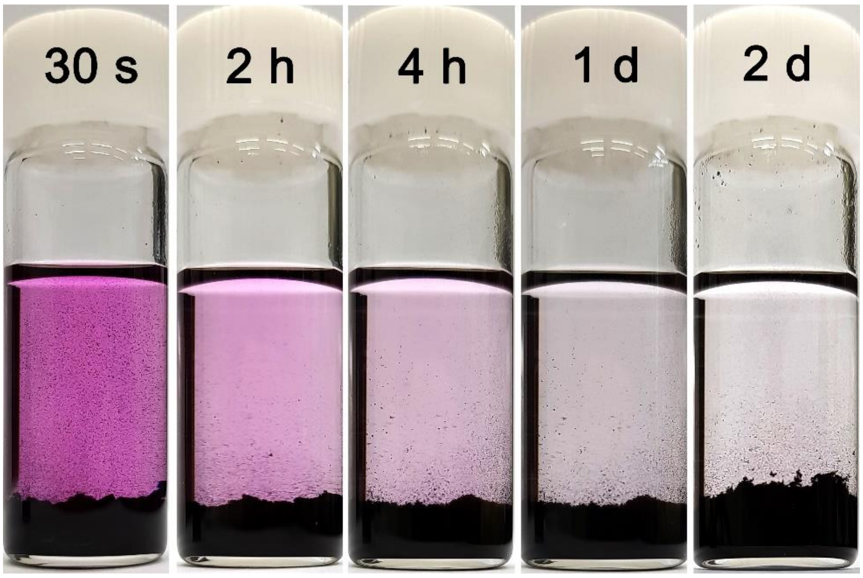

Supplementary Figure 18│Photographs indicating chronicle progress of iodine adsorption when 15 mg of the V2D-BBL structure was immersed in a hexane of I_2 (0.5 mmol, 3 mL).

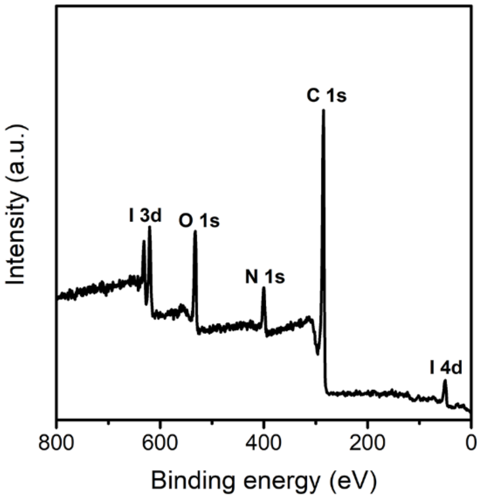

Supplementary Figure 19│XPS survey spectrum of I_2@V2D-BBL structure.

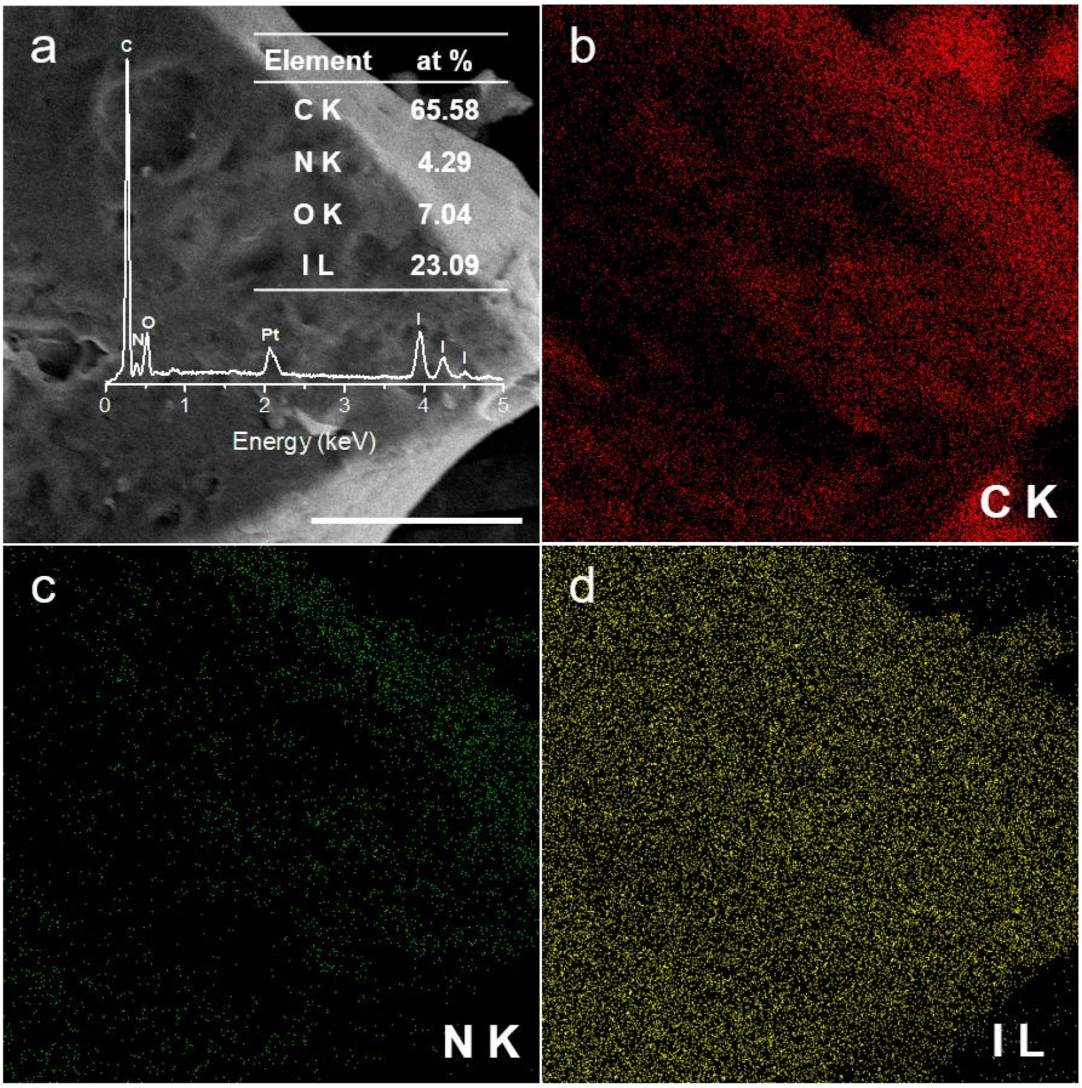

Supplementary Figure 20 | a, EDS spectrum with corresponding table indicating the elemental compositions and SEM elemental mappings of the I_2@V2D-BBL structure complex: **b**, carbon, **c**, nitrogen and **d**, iodine. Scale bar: **a**, 5 μm.

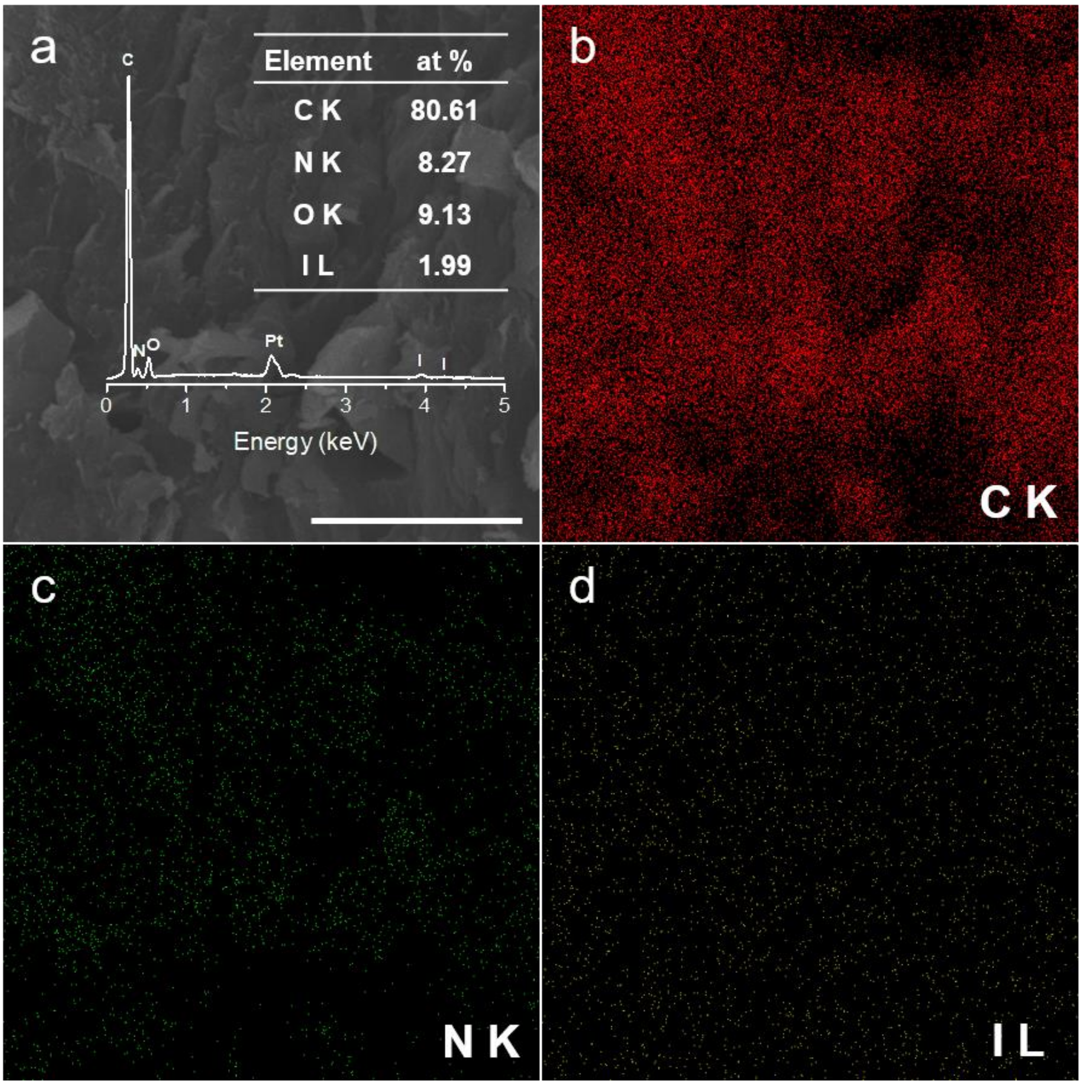

Supplementary Figure 21 | a, EDS spectrum with corresponding table revealing elemental compositions and SEM elemental mappings of the regenerated V2D-BBL structure after 5 reuses: **b**, carbon, **c**, nitrogen and **d**, iodine. Scale bar: **a**, 5 μm.

**Supplementary Table 1 | **Elemental composition of the V2D-BBL structure using different characterization methods

	**C**	**H**	**N**	**O**	**Total**
Theoretical (wt%)	73.00	2.69	12.46	11.86	100
EA (wt %)[a]	72.59	3.76	11.58	12.05	99.8
XPS (wt%)[b]	76.85		11.12	12.03	100
XPS (at%)	80.54		9.99	9.47	100
SEM-EDS (wt%)[c]	72.00		16.17	11.83	100

[a] EA is most reliable technique for elemental counts for bulk sample.

[b, c] XPS and SEM EDS are more sensitive to surface chemical composition.

Supplementary Table 2 | Summaries of H_2, CO_2 and CH_4 uptake of many reported porous organic materials (POMs)

Materials	BET area (m^2 g^{-1})	H_2 uptake (77 K, wt %)	CO_2 uptake (mg g^{-1})	CH_4 uptake (mg g^{-1})	References
**V2D-BBL structure**	**1724**	**1.97 (1.0 bar) 6.03 (110 bar, Total)**	**251 (273 K, 1.0 bar) 1517.5 (270 K, 33 bar, Total) 722 (296 K, 33 bar, Total)**	**24 (273 K, 1.0 bar) 222.5 (270 K, 100 bar, Total) 188 (296 K, 100 bar, Total)**	**This work**
**PIN**	**426**	**1.00 (1.0 bar)**	**105 (273 K, 1.0 bar)**	**9.6 (273 K, 1.0 bar)**	**This work**
2D-BBL-T-HT	615	1.18 (1.0 bar)	160 (273 K, 1.0 bar)	-	Polym. Chem. 2019, 10, 4185-4193
2D-BBL-H-HT	365	1.65 (1.0 bar)	156 (273 K, 1.0 bar)		Polym. Chem. 2019, 10, 4185-4193
COF-1	750	1.46 (35 bar)	230 (298 K, 35 bar)	40 (298 K, 44 bar)	J. Am. Chem. Soc. 2009, 131, 8875
COF-5	1990	0.95 (1.0 bar) 3.54 (35 bar)	870 (298 K, 55 bar)	89 (298 K, 35 bar)	J. Am. Chem. Soc. 2009, 131, 8875
COF-102	3620	0.6 (1.0 bar) 3.6 (35 bar)	1200 (298 K, 55 bar)	187 (298 K, 35 bar)	J. Am. Chem. Soc. 2009, 131, 8875
COF-103	1630	0.6 (1.0 bar) 3.5 (35 bar)	1190 (298 K, 55 bar)	175 (298 K, 35 bar)	J. Am. Chem. Soc. 2009, 131, 8875
Polystyrene	1930	1.4 (1.0 bar)	-	-	Chem. Mater. 2006, 18, 4430
Polyaniline	632	0.96 (1.0 bar)	-	-	J. Mater. Chem. 2007, 17, 4989
Azo-COP-2	729	-	112 (273 K, 1.0 bar)	-	Nat. Commun. 2013, 4, 1357
3D-CON	2247	2.64 (1.0 bar) 5.8 (70 bar)	267 (273 K, 1.0 bar) 750 (298 K, 35 bar)	24 (273 K, 1.0 bar) 237 (298 K, 85 bar)	Angew. Chem. Int. Ed. 2018, 57, 3415
P-PCz	1647	-	245 (273 K, 1.0 bar)	-	Chem. Commun. 2016, 52, 4454
PCTF-1	2235	1.86 (1.0 bar)	143 (273 K, 1.0 bar)	17 (273 K, 1.0 bar)	Chem. Commun. 2013, 49, 3961
PCTF-2	784	0.9 (1.0 bar)	82 (273 K, 1.0 bar)	11 (273 K, 1.0 bar)	Chem. Commun. 2013, 49, 3961
Network-1	1980	1.76 (1.0 bar)	160 (273 K, 1.0 bar)	-	J. Mater. Chem. A. 2014, 2, 8054
Network-A	4077	-	117 (273 K, 1.0 bar)	-	Energy Environ. Sci. 2011, 4, 4239
Network-B	1847	-	145 (273 K, 1.0 bar)	-	Energy Environ. Sci. 2011, 4, 4239
Network-E	1470	-	130 (273 K, 1.0 bar)	-	Energy Environ. Sci. 2011, 4, 4239
Th-1	726	1.11 (1.0 bar)	127 (273 K, 1.0 bar)	-	Adv. Mater. 2012, 24, 5703
PPN-1	827	1.37 (1.0 bar) 3.30 (45 bar)	-	-	Chem. Mater. 2010, 22, 5964
PPN-2	2790	1.51 (1.0 bar)	-	-	Chem. Mater.

		3.76 (40 bar)			2010, 22, 5964
PPN-3	5323	1.58 (1.0 bar)<br>4.28 (42 bar)	-	-	Chem. Mater.<br>2010, 22, 5964
BILP-1	1172	1.9 (1.0 bar)	188 (273 K, 1.0 bar)	23 (273 K, 1.0 bar)	Chem. Mater.<br>2011, 23, 1650
POFs	1063	1.5 (1.0 bar)<br>4.3 (70 bar)	-	-	Chem. Mater.<br>2010, 22, 4974
BILP-10	787	1.6 (1.0 bar)	177 (273 K, 1.0 bar)	16.7 (273 K, 1.0 bar)	J. Mater. Chem.<br>2012, 22, 25409
HPOP-1	1148	1.5 (1.13 bar)	-	-	Macromolecules<br>2011, 44, 5573
HPOP-2	742	1.08(1.13 bar)	-	-	Macromolecules<br>2011, 44, 5573
Cz-POF-1	2065	2.2 (1.0 bar)	202 (273 K, 1.0 bar)	22 (273 K, 1.0 bar)	Chem. Mater.<br>2014, 26, 4023
PCTF-8	625	1.01 (1.0 bar)	111 (273 K, 1.0 bar)	12.2 (273 K, 1.0 bar)	J. Mater. Chem. A.<br>2016, 4, 13450
PECONF-3	851	-	153 (273 K, 1.0 bar)	16 (273 K, 1.0 bar)	Nat. Commun.<br>2011, 2, 401
ILCOF-1	2723	1.3 (1.0 bar)<br>4.7 (40 bar)	60 (273 K, 1.0 bar)<br>1289.5 (298 K, 40 bar)	9 (273 K, 1.0 bar)<br>179.6 (298 K, 40 bar)	Chem. Eur. J.<br>2013, 19, 3324-3328
BDT2	571	1.5 (1.0 bar)	105 (273 K, 1.0 bar)	8 (273 K, 1.0 bar)	ACS Appl. Mater. Interfaces.<br>2016, 8, 27669
BDT3	1010	2.2 (1.0 bar)	165 (273 K, 1.0 bar)	29 (273 K, 1.0 bar)	ACS Appl. Mater. Interfaces.<br>2016, 8, 27669
*p*CTF-1	2034	1.75 (1.0 bar)	219 (273 K, 1.0 bar)	-	Angew. Chem. Int. Ed.<br>2018, 57, 8438
PAF-3	2932	2.07 (1.0 bar)<br>5.5 (80 bar)	153 (273 K, 1.0 bar)	19 (273 K, 1.0 bar)	Energy Environ. Sci.<br>2011, 4, 3991
PAF-4	2246	1.50 (1.0 bar)<br>4.2 (80 bar)	107 (273 K, 1.0 bar)	13 (273 K, 1.0 bar)	Energy Environ. Sci.<br>2011, 4, 3991
CTF-TPC	1668	1.76 (1.0 bar)	188 (273 K, 1.0 bar)	21.5 (273 K, 1.0 bar)	J. Mater. Chem. A.<br>2016, 4, 6259
PSN-1	1045	1.26 (1.0 bar)	150 (273 K, 1.0 bar)	-	Chem. Commun.<br>2014, 50, 1897
NPTN-1	1558	1.44 (1.0 bar)	140 (273 K, 1.0 bar)	-	Macromolecules<br>2014, 47, 2875
TAPOP-1	930	-	154 (273 K, 1.0 bar)	-	RSC Adv.<br>2015, 5, 90135
POP-1	1031	2.78 (60 bar)	-	-	Chem. Commun.<br>2010, 46, 4547
POP-2	1013	2.71 (60 bar)	-	-	Chem. Commun.<br>2010, 46, 4547
POP-3	1246	3.07 (60 bar)	-	-	Chem. Commun.<br>2010, 46, 4547
POP-4	1033	2.35 (60 bar)	-	-	Chem. Commun.<br>2010, 46, 4547
DCX/BCMBP	1904	1.50 (1.0 bar)<br>3.7 (15 bar)	-	70 (36 bar)	Adv. Mater.<br>2008, 20, 1916
BCMBP	1366	1.70 (1.0 bar)<br>2.8 (15 bar)	-	88 (36 bar)	Adv. Mater.<br>2008, 20, 1916
HCP 1	1646	-	75 (298 K, 1.0 bar)	-	J. Mater. Chem.<br>2011, 21, 5475-5483

			585.3 (298 K, 30 bar)		
HCP 2	1684	-	75 (298 K, 1.0 bar)<br>554.5 (298 K, 30 bar)	-	J. Mater. Chem. 2011, 21, 5475-5483
HCP 3	1531	-	70.4 (298 K, 1.0 bar)<br>510.5 (298 K, 30 bar)	-	J. Mater. Chem. 2011, 21, 5475-5483
HCP 4	1642	-	70.4 (298 K, 1.0 bar)<br>466.5 (298 K, 30 bar)	-	J. Mater. Chem. 2011, 21, 5475-5483

**Supplementary Table 3 | **Summary of iodine uptake capacity of many reported porous organic materials (POMs)

Materials	Temperature (°C)	I_2 uptake (g g^{-1})	Maximum time for saturation (h)	References
**V2D-BBL structure**	**75**	**3.00**	**2.5**	**This work**
**PIN**	**75**	**1.80**	**> 24**	**This work**
TPB-MDTP COF	75	6.26	96	Adv. Mater., 2018, 1801991
TTA-TTB COF	75	4.95	96	Adv. Mater., 2018, 1801991
ETTA-TPA COF	75	4.79	72	Adv. Mater., 2018, 1801991
PAF-24	75	2.76	48	Angew. Chem. Int. Ed., 2015, 54, 12733
PAF-23	75	2.71	48	Angew. Chem. Int. Ed., 2015, 54, 12733
PAF-25	75	2.60	48	Angew. Chem. Int. Ed., 2015, 54, 12733
NiP-CMP	75	2.02	24	Chem. Comm., 2014, 50, 8495
AzoPPN	77	2.90	48	Chem. Eur. J., 2016, 22, 11863
Azo-Trip	77	2.38	48	Polym. Chem., 2016, 7, 643
Cg-5P	25	0.87	288	RSC Adv., 2011, 1, 1704
NiMoS chalcogels	60	2.25	48	Chem. Mater., 2015, 27, 2619
CoMoS chalcogels	60	2.00	48	Chem. Mater., 2015, 27, 2619
SbSnS chalcogels	60	2.00	48	Chem. Mater., 2015, 27, 2619
ZnSnS chalcogels	60	2.25	48	Chem. Mater., 2015, 27, 2619
KCoS chalcogels	60	1.60	48	Chem. Mater., 2015, 27, 2619
NTP	75	1.80	48	ACS Macro Lett., 2016, 5, 1039
COF-DL229	75	4.68	24	Chem. Eur. J., 2018. 24, 585
MoSx	60	1.00	24	J. Am. Chem. Soc., 2015, 137, 13943
CX4-NS	75	1.14	8	ACS Appl. Mater. Interfaces., 2018, 10, 17359
BDP-CPP-1	75	2.83	24	J. Mater. Chem. A 2017, 5, 6622
BDP-CPP-2	75	2.23	24	J. Mater. Chem. A 2017, 5, 6622
NBDP-CPP	75	1.50	24	J. Mater. Chem. A 2017, 5, 6622

Supplementary Methods

Preparation of triptycene hexamine (THA) hexahydrochloride[1, 2]: To a stirred solution of the hexakis(diphenylmethylene)triptycene-2,3,6,7,14,15-hexaamine (0.5 g, 0.38 mmol) in tetrahydrofuran (THF, 22 mL), aqueous HCl solution (2.0 M, 1.7 mL, 3.4 mmol) was slowly added. The mixture was stirred at room temperature for 30 min. The precipitated powder was collected by filtration and washed with THF and hexane repeatedly. Then, collected white powder was dried under reduced pressure (0.01 mmHg) to yield quantitative (96 %). The crude product was recrystallized from diluted aqueous HCl solution to afford white needle-type crystals.

Supplementary Note 1 | The C 1s peak can be deconvoluted into four peaks at 284.2, 285.6, 288.5 and 290.7 eV (**Supplementary Figure 3a**). The peaks at 284.2 and 285.6 eV are associated with the sp^2 C–C and sp^2 C–N=C in the aromatic phenyl and benzimidazole rings, respectively, along with the carbonyl C=O peak in pyrrolidinone at 288.5 eV. The satellite peak at 290.7 eV comes from the π-π* transition originating from the extended delocalized electrons[3]. In addition, the O 1s peak can be resolved to two peaks from the dominant carbonyl C=O peak at 531.6 eV and the minor C–O peak at the edges of the structure, and oxygen and/or moisture at 533.1 eV (**Supplementary Figure 3b**).

Supplementary Note 2 | Molecular hydrogen (H_2) is considered a promising energy alternative to replace conventional fossil energy, because of its clean and sustainable nature. Hence, H_2 storage is a crucial global issue for the transition to a hydrogen economy[4]. The standard for on-board H_2 storage systems set by the US Department of Energy (DOE) by the year 2025 is 5.5 wt% and 0.04 kg L^{-1} [5]. The V2D-BBL structure exhibited narrow micropores with remarkable specific surface area, and the H_2 adsorption and desorption isotherms were measured at 77 and 87 K at low-pressure 1.0 bar (**Supplementary Figure 9a**). The isotherm graphs showed completely reversible physisorption without hysteresis. A maximum of 1.97 wt% (9.84 mmol g^{-1}) uptake was observed at 77 K and 1.41 wt% (7.02 mmol g^{-1}) at 87 K. This amount is comparable to many other reported POMs reported in the literature (**Supplementary Table 2**).

To address the pressing global climate change issues of today, a means capturing and storing the CO_2 generated by the combustion of fossil fuels is crucial. Many CO_2 capture technology and materials have been developed. Because of its large surface area and abundant uniform micropores, the V2D-BBL structure also holds huge potential for CO_2 capture. In low-pressure CO_2 uptake experiments, the V2D-BBL structure showed high CO_2 uptake at 1.0 bar, with a value of 25.1 wt% (5.70 mmol g^{-1}) at 273 K and 14.3 wt% (3.26 mmol g^{-1}) at 298 K (**Supplementary Figure 9b**). Moreover, the lack of hysteresis in the CO_2 uptake graph at 1 bar demonstrates a physical interaction occurs between gas molecules and the pore walls of the V2D-BBL structure, which allows the material to regenerate easily without applying heat[1].

Natural gas, mainly methane (CH_4), is considered as an alternative fuel for vehicles, because it is more abundant and economical than gasoline[6]. Accordingly, researchers are looking for efficient methane storage methods and materials. Among the various approaches, POMs are considered good candidates for methane storage because of their environmental stability. To evaluate the CH_4 storage performance of the V2D-BBL structure, CH_4 adsorption and desorption was performed at 273 and 298 K in the low-pressure range (0 − 1 bar). The V2D-BBL structure exhibited high CH_4 uptakes of 2.39 wt% (1.49 mmol g^{-1}) at 273 K and 1.40 wt% (0.87 mmol g^{-1}) at 298 K (**Supplementary Figure 9c**).

Supplementary Note 3 | To investigate the interactions between gases and the V2D-BBL structure, the isosteric heat of adsorption (Q_{st}) for H_2, CO_2 and CH_4 were calculated from the gas adsorption isotherms at two different temperatures, using the Clausius−Clapeyron equation[7]. The Q_{st} for H_2 was measured using H_2 isotherm graphs at 77 and 87 K (**Supplementary Figure 14a**). The Q_{st} of the V2D-BBL structure toward H_2 at zero coverage was found to be 8.51 kJ mol^{-1} and dropped gradually to 6.54 kJ mol^{-1} as the H_2 uptake increased up to 1.97 wt%. The high Q_{st} value in the low-pressure region is attributed to the microporous nature of the material and higher H_2 affinity toward the extended fused aromatic structure with heteroatoms.

Likewise, the Q_{st} values of the V2D-BBL structure toward CO_2 and CH_4 were investigated from the adsorption graphs at 273 and 298 K (**Supplementary Figure 14b, c**). The Q_{st} for CO_2 near the zero coverage was 36.28 kJ mol^{-1} and decreased to 29.11 kJ mol^{-1} at full loading. The high CO_2 uptake with high Q_{st} value can be attributed to the strong interactions between polarizable CO_2 molecules and materials through dipole-quadrupole interactions at nitrogen sites on the benzimidazole ring[4]. Likewise, the Q_{st} toward CH_4 at zero loading was calculated to be 25.12 kJ mol^{-1} and 21.74 kJ mol^{-1} at full coverage. All the recorded Q_{st} values (H_2, CO_2 and CH_4) were higher or comparable to those of other reported POMs and MOFs[8-10].

Supplementary Note 4 | The iodine capturing capability of the V2D-BBL structure was also assessed from solution (**Supplementary Figure 18**). Since there is no dissociative charge transfer between hexane and iodine, hexane was used as the solvent to investigate the interaction between iodine and the V2D-BBL structure[11]. The iodine solution (0.5 mmol, 3 mL) containing 15 mg of the V2D-BBL structure was observed with respect to time for 48 h. The purple colored solution gradually disappeared and became colorless after 48 h. Thus, the V2D-BBL structure with nitrogen rich fused aromatic rings and uniform micropores enhanced iodine capture capacity from both vapor and solution.

Supplementary Note 5 | The morphology of the I_2@V2D-BBL structure complex, and the distribution of iodine, were observed from SEM images and corresponding elemental mappings (**Supplementary Figure 20**). Both the I_2@V2D-BBL structure complex and regenerated V2D-BBL structure (**Supplementary Figure 20-21**) showed unchanged morphologies, suggesting the BBL framework was sufficiently stable for the vapor iodine adsorption/desorption cycles. The amount of iodine was evenly distributed in the I_2@V2D-BBL structure complex, based on the elemental mapping (**Supplementary Figure 20d**) and traces of iodine (chemisorbed) was detected even after regeneration (**Supplementary Figure 21d**), indicating again almost complete removal of iodine from the structure.

References

1 Rabbani, M. G., Reich, T. E., Kassab, R. M., Jackson, K. T. & El-Kaderi, H. M. High CO_2 uptake and selectivity by triptycene-derived benzimidazole-linked polymers. *Chem. Commun.* **48**, 1141-1143 (2012).

2 Mahmood, J. *et al.* A Robust 3D Cage-like Ultramicroporous Network Structure with High Gas-Uptake Capacity. *Angew. Chem. Int. Ed.* **57**, 3415-3420 (2018).

3 Jackson, S. T. & Nuzzo, R. G. Determining hybridization differences for amorphous carbon from the XPS C 1s envelope. *Appl. Surf. Sci.* **90**, 195-203 (1995).

4 Allendorf, M. D. *et al.* An assessment of strategies for the development of solid-state adsorbents for vehicular hydrogen storage. *Energy Environ. Sci.* **11**, 2784-2812 (2018).

5 Park, J. *et al.* A facile synthesis tool of nanoporous carbon for promising H_2, CO_2, and CH_4 sorption capacity and selective gas separation. *J. Mater. Chem. A* **6**, 23087-23100 (2018).

6 He, Y., Zhou, W., Qian, G. & Chen, B. Methane storage in metal–organic frameworks. *Chem. Soc. Rev.* **43**, 5657-5678 (2014).

7 Kloutse, A. F. *et al.* Isosteric heat of hydrogen adsorption on MOFs: comparison between adsorption calorimetry, sorption isosteric method, and analytical models. *Appl. Phys. A* **121**, 2 (2015).

8 Lu, W. *et al.* Porous Polymer Networks: Synthesis, Porosity, and Applications in Gas Storage/Separation. *Chem. Mater.* **22**, 5964-5972 (2010).

9 Dawson, R., Stöckel, E., Holst, J. R., Adams, D. J. & Cooper, A. I. Microporous organic polymers for carbon dioxide capture. *Energy Environ. Sci.* **4**, 4239-4245 (2011).

10 Zhao, D., Yuan, D. & Zhou, H.-C. The current status of hydrogen storage in metal–organic frameworks. *Energy Environ. Sci.* **1**, 222-235 (2008).

11 Li, H., Ding, X. & Han, B.-H. Porous Azo-Bridged Porphyrin–Phthalocyanine Network with High Iodine Capture Capability. *Chem. Eur. J.* **22**, 11863-11868 (2016).
