# Supplementary material for: Vertical two-dimensional layered fused aromatic ladder structure
Source: Nat Commun. 2020 Apr 24;11:2021. doi: 10.1038/s41467-020-16006-0 (PMC7181601; doi:10.1038/s41467-020-16006-0)
Supplement: Supplementary file 3 — Description of Additional Supplementary Files [file 41467_2020_16006_MOESM3_ESM.docx]

Description of Additional Supplementary Files

File Name: Supplementary Video 1

Description: Supplementary Video 1 is showing reversibility of iodine vapor adsorption/desorption for V2D-BBL structure. For regeneration, the I_2_@V2D-BBL structure complex was immersed in ethanol and sonicated for 10 s. Then, no color was observed after filtration, indicating physisorbed I_2_ was removed well.
